# Therapeutic drug monitoring of adalimumab in hidradenitis suppurativa: a prospective observational study

**DOI:** 10.3389/fimmu.2026.1760206

**Published:** 2026-02-05

**Authors:** Stella X. Chen, Bruna Galvao de O. Wafae, Sydney Look-Why, Tracey Otto, Corey Snyder, William Sweet, Nazrin Ashina, Ruby Gibson, Prerna Salian, Maneli Doroudian Tehrani, Thierry Dervieux, Alexa B. Kimball, Martina L. Porter

**Affiliations:** 1 Clinical Laboratory for Epidemiology and Applied Research in Skin (CLEARS), Beth Israel Deaconess Medical Center, Boston, MA, United States; 2 Prometheus Laboratories, San Diego, CA, United States; 3 Harvard Medical School, Boston, MA, United States

**Keywords:** adalimumab, anti-drug antibodies, biologic therapy, drug clearance, hidradenitis suppurativa, pharmacokinetics, therapeutic drug monitoring, tumor necrosis factor-alpha inhibitors

## Abstract

**Background:**

Adalimumab (ADA) is an effective treatment for moderate to severe hidradenitis suppurativa (HS). However, nearly half of patients receiving the standard dose may lose response.

**Objective:**

To establish therapeutic thresholds for HS-specific ADA levels, leading to more personalized treatment.

**Methods:**

We conducted a single-center, prospective observational study of adults with HS treated with ADA (40 or 80mg/week, and 80mg/biweekly) at a specialized HS clinic (January 2023–May 2024). Serum samples were collected after ≥4 weeks of therapy to ensure steady state. Patients were stratified by physician-assessed response (low <50%, partial 50–75%, high >75% improvement). Associations of ADA concentration and clearance with response were tested using Spearman and Pearson correlations; discriminatory performance was evaluated with ROC analysis.

**Results:**

Among 46 enrolled patients, the majority was female (60.9%) and White (54.3%), with a median age of 38 years (IQR: 30–45). ADA serum concentrations positively correlated with clinical response (r = 0.43, p = 0.002). Low responders had significantly lower concentrations than partial (p = 0.029) and high responders (p = 0.007). Clearance was inversely correlated with ADA levels (r = –0.65, p < 0.001). Receiver operating characteristic analysis identified optimal thresholds of 10.7 μg/mL for ADA concentration and 0.5 L/day for clearance.

**Conclusion:**

Higher ADA concentrations and lower drug clearance are associated with better clinical outcomes in HS. ROC analysis identified 10.7 μg/mL (ADA concentration) and 0.5 L/day (clearance) as optimal cut-off values to differentiate low from partial/high responders. These findings suggest that therapeutic drug monitoring may help optimize ADA therapy in HS.

## 1 Introduction

Hidradenitis suppurativa (HS) is a chronic inflammatory disorder of hair follicles, most commonly affecting the axillae, chest, groin and buttocks. Adalimumab (ADA), a Tumor Necrosis Factor-alpha (TNF-α) inhibitor, was the first Food and Drug Administration (FDA)-approved drug for moderate to severe HS. (1) In the seminal PIONEER I and II trials of biologic naïve patients, 42-59% of HS patients treated with ADA 40mg weekly achieved clinical response at 12 weeks. Of those, 45%-52% experienced loss of response by 36 weeks. Moreover, in a follow-up study of the open-label extension period, at least 16% of patients who initially experienced clinical response developed secondary loss of response within 3 years. (2).

While not FDA-approved for HS, infliximab (IFX) is another TNF-α inhibitor frequently used as HS management. Several smaller studies have shown its efficacy in HS, but higher and more frequent dosing than that typically used for inflammatory bowel disease (IBD) are needed to prevent or overcome loss of efficacy. (3, 4) It is unclear why certain patients with HS – whether treated with IFX or ADA – are primary responders, partial responders, or initial responders that develop secondary loss of response. Similar trends are observed in IBD, where roughly thirty percent of patients are primary non-responders to infliximab, and 50% go on to lose response over time. (5, 6) In IBD, the decreased clinical efficacy of these biological medications is associated with inadequate serum drug concentration levels and antibodies to IFX and ADA. (6).

Therapeutic drug monitoring (TDM) of TNF-α inhibitors involves measurement of serum drug concentrations, typically at trough, and anti-drug antibodies. TDM can predict patients who may fail to respond, as well as those who might benefit from dose escalation, thereby improving clinical response rates and prolonging disease control periods. TDM can be conducted either reactively or proactively. (6) Reactive TDM is routinely used in IBD to evaluate biologic serum levels in the setting of treatment failure, inadequate response, or secondary loss of response. (6) Proactive TDM involves correlating clinical disease response to drug concentration levels and anti-drug antibodies at initiation and regular intervals throughout the maintenance dosing period for TNF-alpha inhibitors prior to loss of response. In IBD, several studies have established target drug concentration thresholds in patients to improve clinical outcomes. (6).

TDM has been proposed for HS treatment due to overlapping pathophysiology and therapies with IBD. (3, 7–9) It could extend treatment efficacy, guide dose escalation in suboptimal responders, and identify futility of treatment in the presence of high anti-drug antibody levels so alternative therapies can be considered. However, only small reactive TDM studies in HS exist, and validated ADA thresholds specific to HS have not yet been established, limiting its routine clinical practice. (7) This study aims to identify ADA trough concentrations associated with clinical response among HS patients receiving ADA. We report cross-sectional data to guide the development of targetable ADA thresholds for optimizing therapeutic response in HS.

## 2 Methods

### 2.1 Patient population and study design

This prospective single-center study was initiated at Beth Israel Deaconess Medical Center in January 2023. HS patients aged ≥ 18 years receiving ADA as standard of care were eligible. To minimize inter-rater variability, inclusion required prior clinical evaluation by study investigators prior to starting ADA, though subjects could enroll at any point during their ADA therapy. There were no exclusions for concomitant medications. Participants were recruited from regularly scheduled clinic visits from the HS subspecialty clinics of MLP and ABK. The study was approved by the institutional review board and all participants provided informed consent.

We report results from the first serum samples of patients on ADA therapy from January 2023 to May 2024. A 4-week lead-in period was deemed ideal to ensure that pharmacokinetic steady state had been reached. Maintenance dosages included ADA 40 mg every week, 80 mg every other week (FDA-approved dosing regimens), and off-label dosing of 80 mg weekly.

### 2.2 Procedures and samples

Following informed consent, patient demographics, height, weight, and medical history were obtained. Physician assessments included HS lesion count, Hidradenitis Physician Global Assessment (HS-PGA), and International Hidradenitis Suppurativa Severity Scoring System (IHS4). To prevent bias, blood samples were collected after physician and patient assessments.

ADA serum concentration and anti-adalimumab antibodies (AAA) were measured using homogeneous mobility shift assays from Prometheus Laboratories (San Diego, CA) that does not distinguish neutralizing from non-neutralizing antibodies. The titer cutoff associated with positive antibody to ADA status is set as 1.7 U/mL in this assay format (manufacturer reference). Specimens samples were collected at any time during the inter dose interval (7 or 14 days) owing to the low peak to trough fluctuations at steady state following subcutaneous injection. (10) Lower limit of ADA quantification was <1.6 μg/mL. Drug clearance was calculated using non-linear mixed effect models as described in Wright et al. (11).

### 2.3 Clinical response

Patients were stratified into three physician-assessed clinical response categories based on improvement with ADA: Low responders, Partial responders, and High responders. Predefined clinical criteria were retrospectively applied to participants’ medical records. Whenever available, lesion count prior to or at ADA initiation was used to calculate IHS4; number and intensity of flares were also considered. A flare was defined as an acute worsening documented by the patient and/or clinician that required intervention (e.g., systemic antibiotics, intralesional corticosteroid, incision and drainage, surgery). Low and partial responders demonstrated less than 50% and 50–75% reductions in IHS4, respectively, and/or improved frequency and intensity of flares. High responders exhibited at least a 75% reduction in lesion count and/or experienced rare flares requiring intervention; additionally, patients without reliable baseline lesion counts but with an IHS4 of zero at enrollment were categorized as high responders. Standardized disease outcome scores such as IHS4 could not be calculated for all participants due to missing/unreliable baseline lesion counts for some patients already on maintenance therapy before enrollment. Internal validity of clinical response criteria was assessed through correlation with patient-reported drug response evaluations.

### 2.4 Statistical analysis

The study adhered to the Strengthening the Reporting of Observational Studies in Epidemiology (STROBE) guidelines.(von 12) Statistical analysis was performed using Statistica (version 14.1.0.8) with alpha values set at 0.05 and confidence intervals at 95%. Descriptive statistics were used to compare demographic and clinical characteristics across the three response groups.

Associations between ADA serum concentrations and clearance with clinical response were assessed using Spearman rank test and Pearson correlation, respectively. Wilcoxon-Mann-Whitney test was used to compare median ADA concentrations across groups. Cohen’s kappa evaluated the agreement between patients and physician’s treatment response evaluation.

The discriminatory ability of the ADA serum concentration to predict clinical response was evaluated using Receiver Operating Characteristic (ROC) curve analysis, with the area under the curve (AUC) used as a metric of test performance. For this analysis, the patients were recategorized into “low response” versus “partial plus high response.” Youden’s J statistic was applied to identify the optimal ADA concentration threshold for predicting clinical response.

## 3 Results

### 3.1 Patient characteristics

The study included 49 HS patients, 3 were excluded due to missing physician clinical assessment. Thus, 46 patients were included in the final analysis. The majority of patients were female (60.9%), White (54.3%), with a median age of 38 years old (IQR: 30-45). The mean body mass index (BMI) was 33.3 (SD: 8.5) and 28% were active smokers. Regarding disease severity, 62% were Hurley stage II and 29% were Hurley stage III. Lesion count prior to ADA initiation was available for 87% of cases. Only one patient was concurrently treated with methotrexate at the time of serum sampling. Detailed patient characteristics are depicted in **Table 1**.

**Table 1:** Patient characteristics and therapeutic drug monitoring stratified by response to ADA.

	All patients (n =46)	Low responders (n = 16)	Partial responders (n = 18)	High responders (n = 12)	P value
Age, median (IQR), y	38.0 [30.0, 45.0]	36.5 [29.7, 44.2]	36.5 [30.0, 44.7]	41.5 [36.5, 53.0]	0.387
Female sex, n (%)	28 (60.9)	8 (50.0)	15 (83.3)	5 (41.7)	0.039
Race, n(%)					0.431
White	25 (54.3)	6 (37.5)	10 (55.6)	9 (75.0)	
Black	11 (23.9)	4 (25.0)	5 (27.8)	2 (16.7)	
Asian	4 (8.7)	3 (18.8)	1 (5.6)	0 (0.0)	
Other	6 (13.0)	3 (18.8)	2 (11.1)	1 (8.3)	
Current smoker, n (%)	13 (28.3)	5 (31.2)	2 (11.1)	6 (50.0)	0.065
BMI, mean (SD)	33.28 (8.52)	34.4 (9.1)	33.6 (8.3)	31.1 (8.4)	0.622
Inflammatory bowel disease (IBD)	6 (13)	1 (6.2)	2 (11.1)	3 (25)	0.329
ADA weekly dosage					
20 mg	1 (2.2)	0 (0.0)	1 (5.6)	0 (0.0)	0.644
40 mg	25 (54.3)	8 (50.0)	11 (61.1)	6 (50.0)	
80 mg	20 (43.5)	8 (50.0)	6 (33.3)	6 (50.0)	
IHS4 at ADA initiation*					
Mild	4 (8.7)	0	3 (16.7)	1 (8.3)	0.552
Moderate	18 (39.1)	5 (31.2)	9 (50)	4 (33.3)	
Severe	18 (39.1)	8 (50)	6 (33.3)	4 (33.3)	
Treatment duration, median (IQR), months	13.3 [3.6, 30.2]	4.2 [2.3, 15.4]	14.1 [4.8, 30.2]	30.4 [18.8, 37.4]	0.007
IHS4 at ADA dosing					
Mild	20 (45.5)	3 (20.0)	8 (47.1)	9 (75.0)	0.003
Moderate	16 (36.4)	5 (33.3)	8 (47.1)	3 (25.0)	
Severe	8 (18.2)	7 (46.7)	1 (5.9)	0 (0.0)	
HS-PGA score at ADA dosing					
mild	25 (51.0)	3 (18.8)	9 (50.0)	11 (91.7)	0.014
moderate	15 (30.6)	7 (43.8)	7 (38.9)	1 (8.3)	
severe	2 (4.1)	1 (6.2)	1 (5.6)	0 (0.0)	
very severe	4 (8.2)	4 (25.0)	0 (0.0)	0 (0.0)	
Hurley Stage					
I	4 (8.9)	0 (0.0)	1 (5.9)	3 (25.0)	0.025
II	28 (62.2)	8 (50.0)	14 (82.4)	6 (50.0)	
III	13 (28.9)	8 (50.0)	2 (11.8)	3 (25.0)	
DLQI, median (IQR)	8.0 (3.0-17.0)	17.0 [8.5, 19.0]	6.0 [3.0, 9.0]	6.0 [3.5, 14.0]	0.056
Laboratory values					
ADA concentration (ug/mL), median (IQR)	13.7 [8.6, 22.9]	9.1 [2.7, 14.1]	14.2 [11.3, 22.6]	24.6 [11.2, 33.9]	0.014
ADA Clearance (L/day, median (IQR)	0.5 [0.3-1.0]	0.9 [0.6-1.8]	0.4 [0.3-1.1]	0.4 [0.3-0.5]	0.002
Antibodies, n(%)	10 (21.7)	6 (37.5)	3 (16.7)	1 (8.3)	0.144

ADA, adalimumab; BMI, body mass index (calculated as weight in kilograms divided by height in meters squared); HS-PGA, Hidradenitis Suppurativa Physician Global Assessment; IHS4, International Hidradenitis Suppurativa Severity Score System; IQR: interquartile range; SD: standard deviation.

*Baseline lesion count missing for 6 patients.

Demographics, smoking status, BMI, and weekly ADA dose were similar across the low, partial, and high responder groups. Most high responders had mild disease severity at sample collection, as classified by IHS4 (75%) and HS-PGA (91.7%), aligning with physician-assessed clinical response. Longer ADA treatment duration was observed with greater clinical response, with median durations of 30.4 weeks (IQR: 18.8–37.4) in high responders, 14.1 weeks (IQR: 4.9–30.2) in partial responders, and 4.24 weeks (IQR: 2.3–15.4) in low responders.

Patient assessments of treatment response aligned with physician ratings in 63% of cases across all three response categories, with Cohen’s kappa indicating moderate agreement (κ = 0.44, p<0.001). When responses were grouped as “low response” versus “partial plus high response,” as applied in the ROC analysis, agreement increased to 80.4%, with a kappa coefficient of 0.59 (p<0.001), also reflecting moderate agreement.

### 3.2 ADA drug concentration and clearance

**Figure 1:**
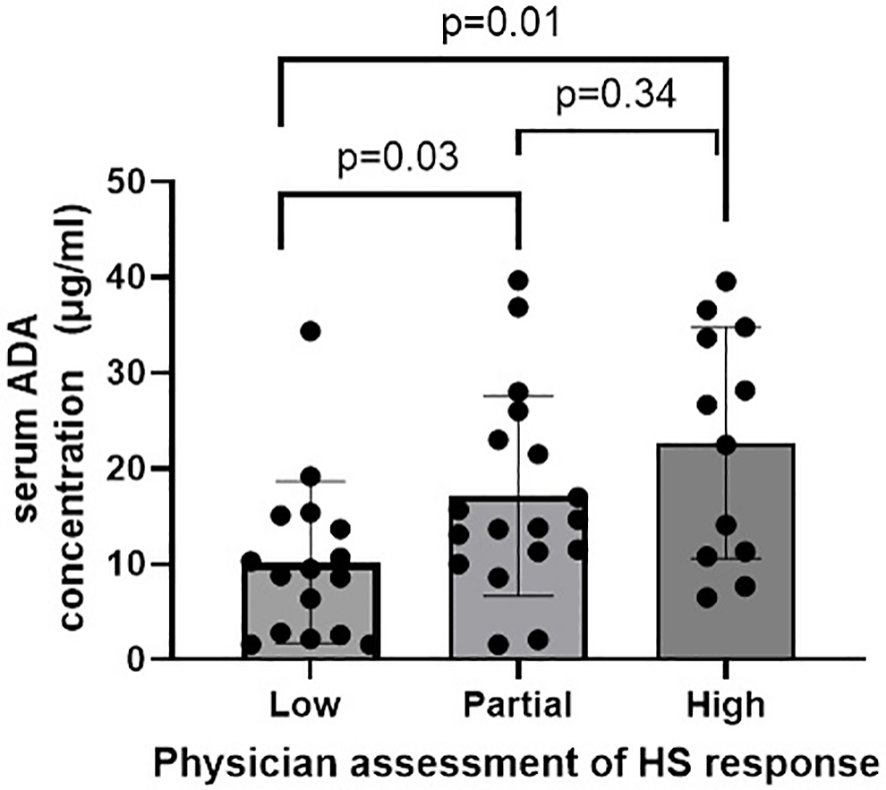
ADA levels for low, partial and high responders. Median IQR ADA concentration for low partial and high physician assessment of HS response was 9.15 [2.68-14.52], 14.25 [11.19-23.25] and 24.60 [11.01-34.34] L/day, respectively.

ADA serum levels ranged from 1.6 to 39.7 ug/mL, with a median concentration of 13.7 ug/mL. A positive correlation was observed between ADA levels and clinical response (r=0.43, p=0.002). Low responders had significantly lower median ADA concentration (9.1 ug/mL) compared to partial responders (14.2 ug/mL; p=0.029) and high responders (24.6 ug/mL; p=0.007). The difference between partial and high responders was not statistically significant (p=0.34) (**Figure 1**).

**Figure 2:**
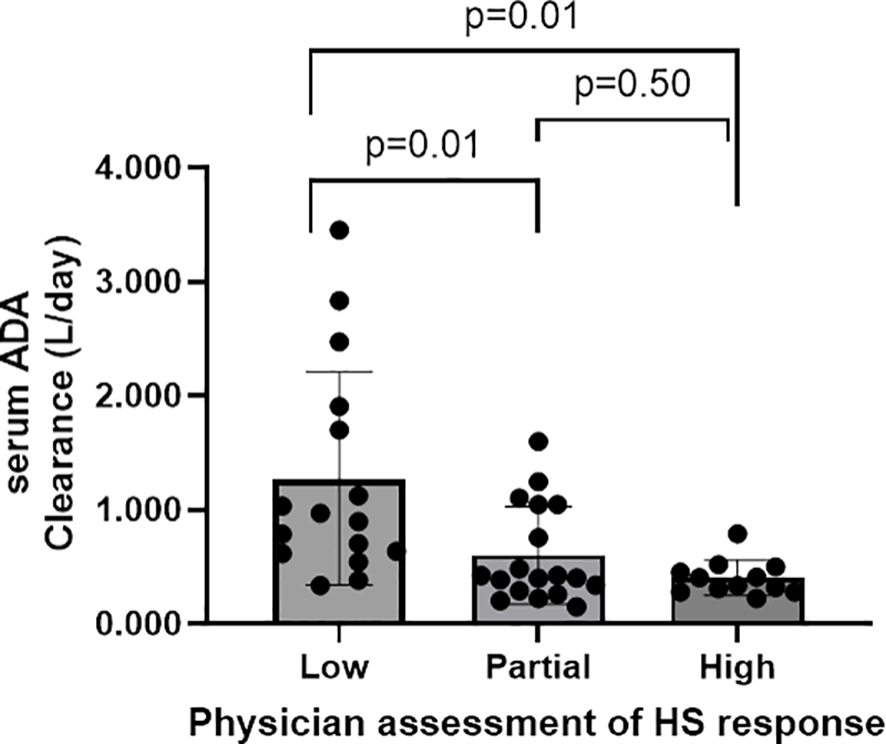
ADA Clearance for low, partial and high responders. Median IQR ADA Clearance for low partial and high physician assessment of HS response was 0.936 [0.627-1.822], 0.415 [0.290-1.05] and 0.373 [0.295-0.482] L/day, respectively.

A significant negative correlation was found between ADA concentration and drug clearance, (r=-0.65 (p<0.001). Low responders had a significantly higher median ADA clearance of 0.94 L/day compared to partial responders (0.41 L/day; p = 0.012) and high responders (0.37 L/day; p < 0.001) (**Figure 2**).

Antibodies against ADA were detected in 21.7% of patients. Although more common in low responders (37.5%) than in partial (16.7%) and high responder (8.3%), the difference was not statistically significant (p=0.144).

### 3.3 ADA concentration threshold

**Figure 3:**
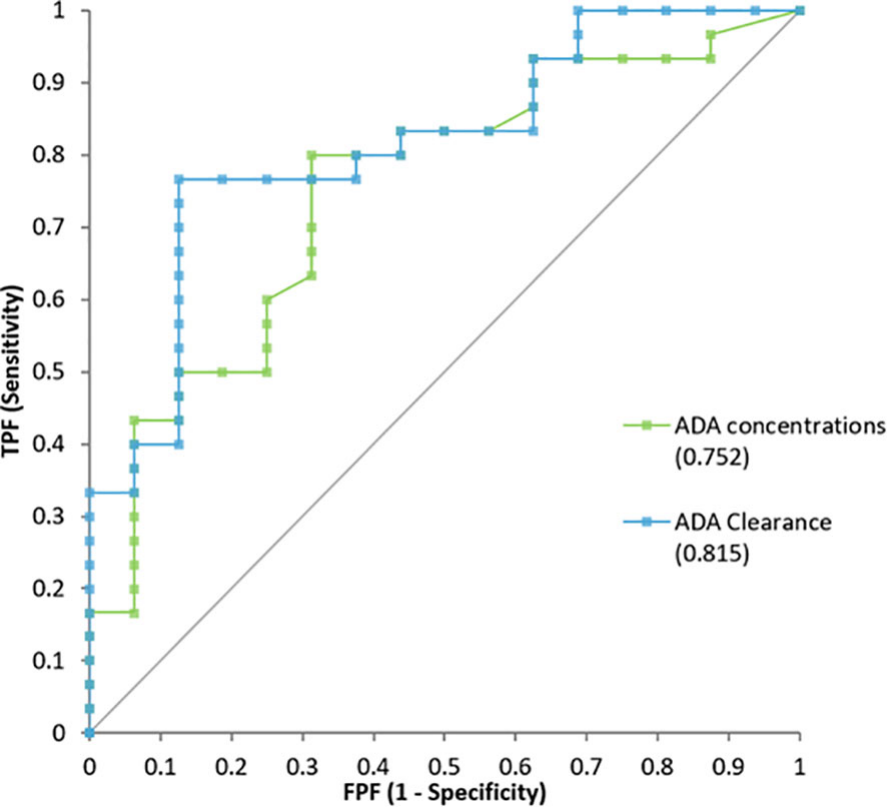
Receiver Operator analysis for ADA concentration and ADA clearance.

**Figure 4:**
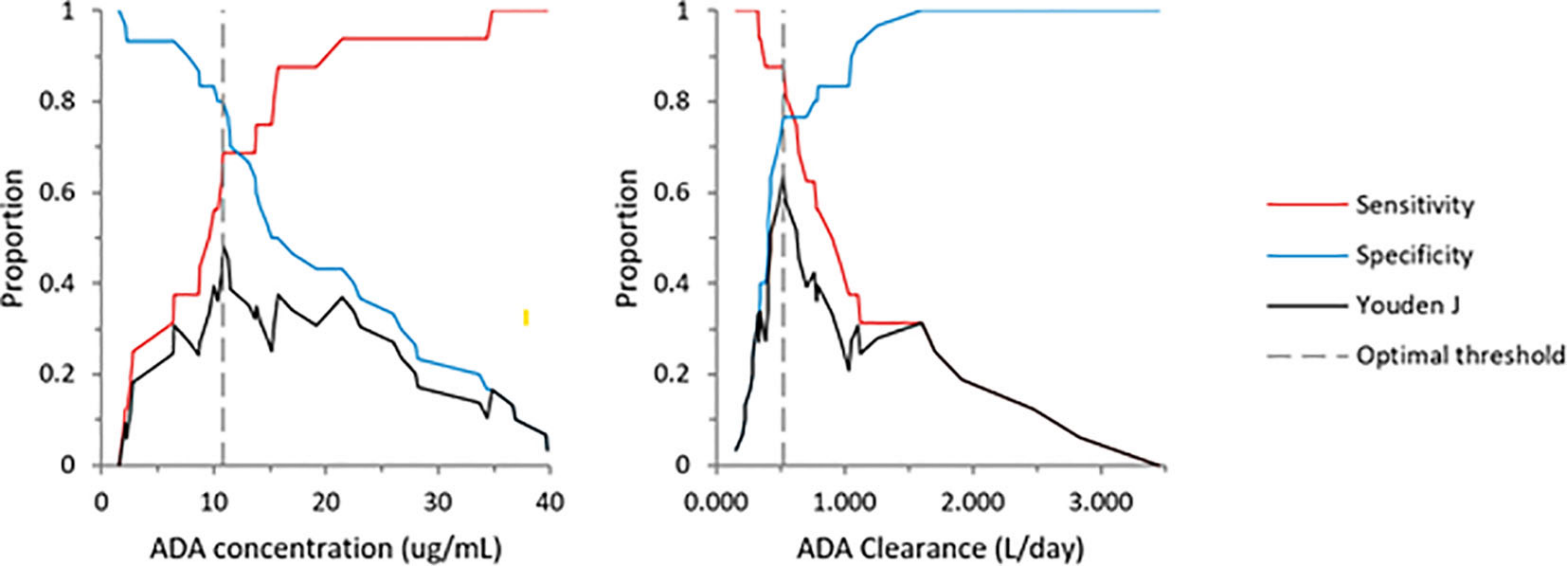
Cutoff values for ADA concentration and Clearance in relation to low *vs* partial and high physician assessment of HS response. Left panel: ADA concentration: 10.7 µg/mL; right panel: Clearance: 0.543 L/day.

ADA concentration and clearance effectively differentiated low responders from partial and high responders. The area under the curve (AUC) was 0.752 (95% CI: 0.60–0.90) for ADA concentration and 0.815 (95% CI: 0.69–0.94) for clearance (**Figure 3**). The optimal ADA concentration threshold was 10.7 μg/mL, with 80% sensitivity and 69% specificity for distinguishing low from partial/high responders. For ADA clearance, the optimal cut-off was 0.5 L/day, with 77% sensitivity and 87% specificity (**Figure 4**). When dichotomizing ADA concentration at 10.7 µg/mL, 82.8% patients with ADA ≥10.7 µg/mL achieved partial/high response, compared with 35.3% patients with ADA <10.7 µg/mL (Fisher’s exact p=0.003).

## 4 Discussion

In this prospective therapeutic drug monitoring study in the HS population, higher adalimumab concentrations and lower clearance were associated with better clinical response. ROC analysis identified 10.7 µg/mL (concentration) and 0.5 L/day (clearance) as cut-offs distinguishing low from partial/high responders.

In psoriasis, optimal response to ADA has been linked to trough levels of 3.5-7.0 µg/mL, with a minimally effective level of 3.2 µg/mL distinguishing responders from nonresponders. (13, 14) In IBD, maintenance ADA levels are typically targeted to at least 8-12 µg/mL. (6) In contrast, our study suggests higher ADA drug levels are needed for HS compared to other inflammatory disorders, with high responders having a median level of 24.6 µg/mL. In the pivotal phase 3 PIONEER HS, mean ADA serum concentration was approximately 9 µg/mL at week 12, with response rates ranging 42-59%. (15) Of note, these trials used the HiSCR50 threshold, while our study aimed at higher lesion reductions to be considered high response. *Post-hoc* analysis of the integrated PIONEER data found 50.6% achieving HiSCR 50, 30.1% achieving HiSCR 75, and 15.2% achieving HiSCR 90 for the ADA 40mg weekly group. (16).

In our clinical practice, patients with inadequate response to ADA 40 mg weekly are typically escalated to 80 mg weekly. Some patients with severe disease may continue on ADA, despite only partial benefit, due to contraindications, comorbidities, or insurance limitations preventing a switch to alternative therapies. Notably, despite similar dosing distributions across response groups, serum concentrations differed, suggesting factors beyond administered dose contribute to exposure variability. Obesity may contribute to higher clearance and greater inflammatory burden in HS, potentially increasing exposure requirements. A recent retrospective HS case series found that normal-weight patients had significantly higher adalimumab concentrations than overweight patients. (17–19) However, more studies are needed do further understand the factors influencing ADA levels in the HS population.

In the IBD literature, the development of AAA can lower free drug levels and neutralize biologic therapy. (6) A meta-analysis by Bots et al. found that 7.5% of IBD patients treated with ADA developed AAA. (20) A systematic review of published trials reported that 7-11% of 418 HS patients treated with ADA developed AAA. (21) In the PIONEER studies, no patients with antibodies achieved primary endpoint, though patients were only tested for antibodies if ADA concentrations were below 2 µg/mL. (15) In our study, 21.7% of our cohort developed AAA, the majority in the low responder group, however the difference was not statistically significant. (37.5% in low responders, 16.7% in partial responders, and 8.3% in high responder, p=0.144). Notably, secukinumab, another FDA approved therapy for moderate to severe HS demonstrated low immunogenicity (<0.1%) in its phase III clinical trials. (22).

In our study, ADA serum concentration predicted clinical response, with a minimum concentration of 10.7 µg/mL distinguishing low responders from partial/high responders. This highlights a role for reactive TDM, in which patients with inadequate response or loss of response can be tested for ADA levels and AAA. Clearance (CL) was a more reliable predictor of clinical response than ADA concentration in our study, consistent with studies in IBD. (11) However, using CL routinely in managing HS patients may be more challenging to implement and interpret.

In addition to the clinical benefits, two systematic reviews have demonstrated that TDM with anti-TNFs is cost effective in both IBD and RA, however the majority of included studies included only infliximab which has higher costs than ADA. Therefore, future research should evaluate the cost-effectiveness specific to the HS population (23, 24).

A small subset of AAA-negative patients had low response despite high drug levels, consistent with pharmacodynamic/primary nonresponse and highlighting TDM’s role in avoiding prolonged ineffective therapy. In partial responders and low drug levels, higher off-label ADA dosing can be considered. In patients with AAA, low doses of methotrexate or azathioprine may be added, as is routinely done in IBD (6). Methotrexate can decrease both anti-drug antibody development and reduces drug clearance. (25).

Our study reflects real-world clinical practice, incorporating not only the lesion count, as well as flare frequency and severity as additional criteria for evaluating response, aligning with methodologies used in previous research (26). This was necessary due to missing and less reliable lesion count at time of ADA initiation, representing the main limitation in our study. Nonetheless, it’s crucial that these findings are validated in a prospective, multicenter study employing standardized outcomes. Other limitations of this study include the small sample size and single-center design.

## 5 Conclusion

Our study suggests that higher serum ADA concentrations are associated with better clinical responses in patients with HS. Despite similar demographic profiles and ADA dosing regimens across low, partial, and high responder groups, low responders had significantly lower ADA levels, while high responders achieved nearly double the median ADA concentration. Furthermore, lower drug clearance was strongly correlated with improved clinical outcomes. Receiver operating characteristic (ROC) analysis identified 10.7 µg/mL as the optimal serum ADA concentration cut-off for at least a partial response, but the median level of 24.6 µg/mL in the high responders may suggest that higher doses might be required. A clearance threshold of 0.5 L/day distinguished low responders from partial/high responders. Together, these findings highlight the potential of TDM to optimize ADA therapy for HS.

## Data Availability

The raw data supporting the conclusions of this article will be made available by the authors, without undue reservation.
